# Effect of Thiopurine on Potential Surgical Intervention in Crohn’s Disease in Korea: Results from the CONNECT Study

**DOI:** 10.3390/jcm10010025

**Published:** 2020-12-23

**Authors:** Hee Man Kim, Jin Woo Kim, Hyun-Soo Kim, Joo Sung Kim, You Sun Kim, Jae Hee Cheon, Won Ho Kim, Byong Duk Ye, Won Moon, Sung Hee Jung, Young-Ho Kim, Dong Soo Han

**Affiliations:** 1 Department of Internal Medicine, Yonsei University Wonju College of Medicine, Wonju 26426, Korea; loverkorea2009@gmail.com (H.M.K.); bluebloood@naver.com (J.W.K.); 2 Department of Internal Medicine, Liver Research Institute, Seoul National University College of Medicine, Seoul 03080, Korea; jooskim@snu.ac.kr; 3 Department of Internal Medicine, Seoul Paik Hospital, Inje University College of Medicine, Seoul 04551, Korea; yousunk69@korea.com; 4 Department of Internal Medicine, Institute of Gastroenterology, Yonsei University College of Medicine, Seoul 03722, Korea; geniushee@yuhs.ac (J.H.C.); kimwonho@yuhs.ac (W.H.K.); 5 Asan Medical Center, Department of Gastroenterology, University of Ulsan College of Medicine, Seoul 05505, Korea; bdye@amc.seoul.kr; 6 Department of Internal Medicine, Kosin University College of Medicine, Busan 49267, Korea; moonone70@hanmail.net; 7 Department of Internal Medicine, Eulji University College of Medicine, Eulji University Hospital, Daejeon 34824, Korea; jsh@eulji.ac.kr; 8 Samsung Medical Center, Department of Internal Medicine, Sungkyunkwan University School of Medicine, Seoul 03181, Korea; younghokim@skku.edu; 9 Department of Internal Medicine, Hanyang University College of Medicine, Guri 11923, Korea; hands@hanyang.ac.kr

**Keywords:** Crohn’s disease, surgery, azathioprine, TNF-α antagonist

## Abstract

**Objectives:** The incidence of Crohn’s disease and the number of associated surgeries are increasing in Korea. This study investigated the effect of azathioprine/6-mercaptopurine (6-MP) and TNF-α antagonists on abdominal and perianal surgery in Korean patients with Crohn’s disease. **Design:** A retrospective cohort study. **Setting:** Data from the Crohn’s Disease Clinical Network and Cohort (CONNECT) were used. Patients with confirmed Crohn’s disease between 1982 and 2008 from 32 hospitals in the Republic of Korea were enrolled. The effect of azathioprine/6-MP on abdominal and perianal surgery was analysed using logistic regression analysis adjusting for age and sex. **Participants:** In total, 1161 Crohn’s disease patients were included in the Republic of Korea in the surgery (*n* = 462, male = 339, female = 123) and control groups (*n* = 699, male = 484, female = 215). **Results:** In total, 1161 patients were selected, with 462 patients who underwent abdominal (*n* = 245) or perianal surgery (*n* = 217). The preoperative usage rates of azathioprine/6-MP were 18.8% and 65.1% (*p* < 0.0001) in the surgery and control groups, respectively. The preoperative usage rates of TNF-α antagonists were 7.1% and 23.3% (*p* < 0.0001) in the surgery and control groups, respectively. A multivariate analysis revealed that the preoperative use of azathioprine/6-MP had an odds ratio of 0.094 for all surgeries (95% confidence interval [CI]: 0.070–0.127, *p* < 0.0001), 0.131 for abdominal surgery (95% CI: 397–1.599, *p* < 0.0001), and 0.059 for perianal surgery (95% CI: 0.038–0.091, *p* < 0.0001). The preoperative use of TNF-α antagonists had an odds ratio of 0.225 for all surgeries (95% CI: 0.151–0.335, *p* < 0.0001), 0.403 for abdominal surgery (95% CI: 0.261–0.623, *p* < 0.0001), and 0.064 for perianal surgery (95% CI: 0.026–0.160, *p* < 0.001). **Strengths of this study:** The study presents new evidence of the reduced risk of surgery following azathioprine use in Crohn’s disease patients. **Limitations of this study** (1) This was not a controlled prospective study. (2) There was a selection bias specific to the CONNECT cohort. (3) The combination or sequential use of azathioprine/6-MP and TNF-α antagonists was not excluded. **Conclusion:** Azathioprine/6-MP is significantly associated with a reduced risk of abdominal and perianal surgery in Korean patients with Crohn’s disease.

## 1. Introduction

Crohn’s disease (CD) is a chronic inflammatory disease that mainly affects the gastrointestinal (GI) tract. It is caused by the dysregulation of immune responses and can eventually lead to structural damage and complications in the GI tract that require surgical intervention or extensive medical therapies. Over the last few decades, the incidence of CD has been increasing globally, including in East Asia and Korea, where its clinical features are slightly distinct from those in Western countries [1,2]. In addition, as the incidence of CD has increased, the annual number of surgeries in CD patients has simultaneously increased from 597 in 2009 to 857 in 2015 in Korea, according to data from the Korea Health Insurance Review and Assessment Service [3]. The main types of surgery associated with CD are colorectal (31.2%), small bowel (29.4%), and anal (39.4%) surgery [3].

Immunomodulators such as azathioprine and 6-mercaptopurine (6-MP) facilitate the maintenance of CD remission, reducing the risk of surgery and decreasing postoperative recurrence in patients; however, the effectiveness of the early initiation of azathioprine remains controversial [1,2,3,4]. Recently, biologic agents, including anti-Tumour Necrosis Factor (TNF)-α antibodies, have exhibited promising improvements in inducing remission in patients, though responsiveness to such treatments diminishes over time [1,4,5,6].

To the best of our knowledge, no cohort studies have investigated the efficacy of medical therapies, including azathioprine and anti-TNF-α agents, on the prevention or risk reduction of surgery in Korean patients with CD [7]. Therefore, the present study investigated whether such medical therapies are associated with reduced surgery risk in Korean patients using a retrospective-prospective cohort in Korea, the Crohn’s disease Clinical Network and Cohort (CONNECT). We investigated the effect of azathioprine/6-MP and TNF-α antagonists on abdominal and perianal surgery in Korean patients with CD.

## 2. Materials and Methods

### 2.1. Study Population

In this study, we obtained data from CONNECT, in which patients who had a confirmed CD diagnosis between 1982 and 2008 in 32 Korean tertiary referral hospitals were registered. A total of 1387 patients with CD were analysed. Patients who underwent surgery unrelated to CD, such as definite appendicitis and total hip replacement, were classified as having not had surgery. Patients with missing medication data were excluded. The institutional review boards of all 32 hospitals approved the cohort registry and our studies. The requirement of written informed consent was waived because the study was based on a review of medical records.

The patients were classified into two groups: surgery and control groups (no surgery). Patients in the surgery group were further classified into abdominal and perianal surgery subgroups. CD with perianal involvement was considered to have a clinical course distinct from that of CD without perianal involvement [8]. The abdominal surgery subgroup was composed of patients who had abdominal surgery due to the gastrointestinal tract complications of CD, such as intestinal stricture, perforation, and intraabdominal abscess. Conversely, the perianal surgery subgroup consisted of patients who had perianal surgery due to perianal CD, such as perianal fistula, ulcer, abscess, and stricture [9]. The control group, as a reference group, consisted of patients without a history of surgery or those with a history of surgery unrelated to CD. The use of two types of therapy, azathioprine/6-MP and TNF-α antagonists, was investigated in all patients. TNF-α antagonists included infliximab and adalimumab. The preoperative use of azathioprine/6-MP and TNF-α antagonists was analysed retrospectively among patients who underwent surgery. Eventually, 462 (39.8%) patients who underwent surgery were selected, and 699 (60.2%) patients without surgery were defined as the control group.

### 2.2. Statistical Analysis

The endpoints of azathioprine/6-MP and TNF-α antagonist treatment were abdominal and perianal surgery related to CD. Therefore, postoperative use of the therapies was considered no usage or similar to no usage in the control group, whereas preoperative use of the therapies was considered to use or similar to use in the control group.

The effects of azathioprine/6-MP and TNF-α antagonists on abdominal and perianal surgery were analysed using logistic regression analysis adjusting for age and sex.

## 3. Results

### 3.1. Characteristics of Study Population

Among the 1161 patients, 542 (46.7%) received azathioprine/6-MP therapy. The preoperative usage rates of azathioprine/6-MP were 18.8% and 65.1% in the surgery (*n* = 462) and control groups (*n* = 699), respectively (*p* < 0.0001; Table 1), whereas the preoperative usage rates of azathioprine/6-MP were 22.9% and 14.3% in the abdominal and perianal surgery subgroups, respectively (*p* < 0.0001).

Among the 1161 patients, 196 (16.9%) received TNF-α antagonists. The preoperative usage rates of TNF-α antagonists were 7.1% and 23.3% in the surgery (*n* = 462) and control (*n* = 699) groups, respectively (*p* < 0.0001; Table 1), whereas the preoperative usage rates of azathioprine/6-MP were 11.4% and 2.3% in the abdominal and perianal surgery subgroups, respectively (*p* < 0.0001).

### 3.2. Univariate Analysis

Out of the 542 patients who used azathioprine/6-MP, 87 (16.1%) patients underwent surgery (Table 2). Age and sex were not different between the patients with or without surgery (*p* = 0.797 and 0.497, respectively). Among the 87 patients who underwent surgery, the mean time to the first use of azathioprine/6-MP from diagnosis was 26.55 ± 36.20 months, which was not significantly different from that for patients who did not undergo surgery (33.01 ± 40.59 months, *p* = 0.195). The mean time to surgery from the first use of azathioprine/6-MP was 31.93 ± 32.96 months.

Out of the 196 patients who used TNF-α antagonists, 33 (16.8%) received surgery (Table 2). Age and sex were not different between the patients with or without surgery (*p* = 0.840 and 0.404, respectively). Among the 33 patients who underwent surgery, the mean time to the first use of TNF-α antagonists from diagnosis was 50.00 ± 40.52 months, which was not significantly different from that in patients who did not undergo surgery (64.45 ± 47.42 months, *p* = 0.104). The mean time to surgery from the first use of TNF-α antagonists was 22.03 ± 21.23 months.

### 3.3. Multivariate Analysis

In the multivariate analysis, the odds ratios of azathioprine/6-MP were 0.094 (95% confidence interval [CI]: 0.070–0.127, *p* < 0.0001) for all surgeries, 0.131 (95% CI: 0.092–0.186, *p* < 0.0001) for abdominal surgery, and 0.059 (95% CI: 0.059–0.091, *p* < 0.0001) for perianal surgery (Table 3). The odds ratios of TNF-α antagonists were 0.225 (95% CI: 0.151–0.335, *p* < 0.0001) for all surgeries, 0.403 (95% CI: 0.261–0.623, *p* < 0.0001) for abdominal surgery, and 0.064 (95% CI: 0.026–0.160, *p* < 0.0001) for perianal surgery (Table 4).

## 4. Discussion

This study showed that azathioprine/6-MP and TNF-α antagonists are associated with low risks of abdominal and perianal surgery in Korean patients with CD.

Immunomodulators such as azathioprine and 6-MP are useful for the maintenance of remission and the prevention of the postoperative recurrence of CD [10,11]. In addition, azathioprine is associated with a low risk of surgical intestinal reTable in patients [10]. However, controversy has persisted over the efficacy of azathioprine. One population-based study reported that the early initiation of thiopurines had no additional surgery reduction benefit in CD patients [12]. In addition, a randomised controlled trial in newly diagnosed CD patients showed that azathioprine had no benefits with regard to steroid-free remission or relapse rates, compared to placebo [13]. In our study, azathioprine/6-MP exhibited a 92% reduction in surgery risk.

TNF-α antagonists such as infliximab and adalimumab facilitate the induction and maintenance of the remission of CD. In addition, TNF-α antagonists have been observed to reduce hospitalisation and surgery, compared to placebo [14]. In the present study, TNF-α antagonists exhibited an 84% reduction in surgery risk. The patients in this study were enrolled between 1982 and 2010, and since 2006, infliximab has been covered by National Insurance; however, it should be administered in a stepwise manner. The usage rate of azathioprine/6-MP was much higher than that of anti-TNF-α therapy (Table 1). This could be one of the factors contributing to the higher odds ratios in azathioprine/6-MP than in TNF-α antagonists (Table 3).

Perianal involvement of CD is a common manifestation, ranging from 40% to 90%, in patients with large bowel involvement [15]. Azathioprine/6-MP is effective in treating perianal fistulas of patients with CD [16]. Anti-TNF-α therapy improves the symptoms of perianal CD, though complete remission is rare [6]. Our study shows that azathioprine/6-MP and anti-TNF-α therapy are very effective in reducing the risk of surgery in patients with perianal CD.

According to data from the Korean Health Insurance and Review Agency and the Korean National Health Insurance, the overall usage rates of immunomodulators (azathioprine or 6-MP) and TNF-α antagonists (infliximab or adalimumab) were 66.9% and 19.6%, respectively, in 8974 patients with CD, between 2010 and 2016 [17]. In our study, 46.4% of the patients received azathioprine/6-MP therapy and 14.8% of the patients received anti-TNF-α therapy, between 1982 and 2010. The usage rates of immunomodulators and TNF-α antagonists have increased over time.

The present study had some limitations. First, this study was not a controlled prospective study. The analyses in our study were retrospective so that missing and unmatched data were omitted. We should have had more targeted data to minimise faults and biases. However, since the incidence of CD is very low, considerable effort and time are required to undertake qualified prospective studies. Consequently, we consider it necessary to pass on the knowledge to the public more rapidly rather than to wait for the collection of more data. Second, there was a selection bias specific to the CONNECT cohort. In one study using data from the Korean National Health Insurance claims database between 2010 and 2014, a total of 57,286 patients with CD were identified, out of whom 3731 (6.5%) patients had received anti-TNF-α therapy [18]. In our study, 196 (16.9%) patients among the 1161 patients had received anti-TNF-α therapy. The higher rate of anti-TNF-α therapy in our study might be due to selection bias because the data of patients in the CONNECT registry were collected from tertiary referral hospitals, where the conditions of the referred patients are expected to be more severe than those of patients in other hospitals. Third, some variables were lacking or were uncontrolled, including disease characteristics (age at diagnosis, behaviour, location, disease extension), diagnostic modalities (radiologic or endoscopic), years of evolution from diagnosis of the disease to surgery, or smoking. Although such factors could have influenced our findings, the present study was a retrospective study. Fourth, the cohort data did not distinguish medication non-users and users who stopped medication owing to side effects, who could also be at an increased risk of perianal and/or abdominal surgery. Finally, the combination or sequential use of azathioprine/6-MP and TNF-α antagonists was not excluded.

In conclusion, azathioprine/6-MP is strongly associated with low abdominal and perianal surgery risk in CD patients in Korea. However, the retrospective study design had some limitations, and prospective interventional clinical trials are necessary to evaluate the effects of medical treatment on surgery in Korean patients with CD.

## Figures and Tables

**Table 1:** Clinical features of the patients with Crohn’s disease (CD) (*n* = 1161).

	CD-Related Surgery			No Surgery	*p* Value		
	Total	Abdominal	Perianal	(Control)	Surgery vs. No Surgery	Abdominal Surgery vs. No Surgery	Perianal Surgery vs. No Surgery
***n***	462 (39.8%)	245	217	699 (60.2%)			
Age at diagnosis (years)	25.23 ± 9.63	27.38 ± 10.76	22.70 ± 7.18	28.36 ± 13.27	<0.0001	0.333	<0.0001
Sex					0.129	0.939	0.010
male	339 (73.4%)	169 (69.0%)	170 (78.3%)	484 (69.2%)			
Female	123 (29.1%)	76 (31.0%)	47 (21.7%)	215 (30.8%)			
Location at diagnosis					0.111	-	-
L1	58 (12.6%)	41 (16.7%)	17 (7.8%)	119 (17.0%)			
L2	59 (12.8%)	25 (10.2%)	34 (15.7%)	92 (13.2%)			
L3	153 (33.1%)	67 (27.3%)	86 (39.6%)	201 (56.8%)			
L4	10 (2.2%)	7 (2.9%)	3 (1.4%)	6 (0.9%)			
L3 + 4	1 (0.2%)	0 (0%)	1 (0.5%)	2 (0.3%)			
Unknown	181 (39.2%)	105 (42.9%)	76 (35.0%)	279 (39.9%)			
Behaviour at diagnosis					<0.0001	-	-
B1	156 (33.8%)	61 (24.9%)	95 (43.8%)	286 (40.9%)			
B2	37 (8.0%)	30 (12.2%)	7 (3.2%)	42 (6.0%)			
B3	63 (13.6%)	39 (15.9%)	24 (11.1%)	43 (6.2%)			
Unknown	206 (44.6%)	115 (46.9%)	91 (41.9%)	328 (46.9%)			
AZA/6-MP					<0.0001	<0.0001	<0.0001
Preoperative usage	87 (18.8%)	56 (22.9%)	31 (14.3%)	455 (65.1%)			
Postoperative or no usage	375 (81.2%)	189 (77.1%)	186 (85.7%)	244 (34.9%)			
TNF-α antagonists					<0.0001	<0.0001	<0.0001
Preoperative usage	33 (7.1%)	28 (11.4%)	5 (2.3%)	163 (23.3%)			
Postoperative or no usage	429 (92.9%)	217 (88.6%)	212 (98.2%)	536 (76.7%)			

L1, terminal ileum; L2, colon; L3, ileocolon; L4, upper GI; B1, non-structuring, nonpenetrating; B2, structuring; B3, penetrating; AZA/6-MP, azathioprine/6-mercaptopurine.

**Table 2:** Clinical features of the CD patients with the use of medicine.

Medicine	*n*	Preoperative Usage		*p* Value
		Surgery	No Surgery	Surgery vs. No surgery
**AZA/6-MP**	542	87 (16.1%)	455 (83.9%)	
Age at diagnosis (years)		23.10 ± 8.60	25.28 ± 10.90	0.797
Sex (male)		59 (67.8%)	325 (71.4%)	0.497
Time from diagnosis to usage (months)		26.55 ± 36.20	33.01 ± 40.59	0.195
Time from usage to surgery (months)		31.93 ± 32.96		
Location at diagnosis				0.038
L1		6 (6.9%)	56 (12.3%)	
L2		15 (17.2%)	63 (13.8%)	
L3		36 (41.4%)	145 (31.9%)	
L4		3 (3.4%)	3 (0.7%)	
L3 + 4		0 (0%)	2 (0.4%)	
Unknown		27 (31.0%)	186 (40.9%)	
Behaviour at diagnosis				0.318
B1		37 (42.5%)	179 (39.3%)	
B2		8 (9.2%)	33 (7.3%)	
B3		9 (10.3%)	29 (6.4%)	
Unknown		33 (37.9%)	214 (47.0%)	
**TNF-α antagonists**	196	33 (16.8%)	163 (83.2%)	
Age at diagnosis (years)		22.64 ± 10.10	24.18 ± 9.53	0.840
Sex (men)		20 (60.6%)	111 (68.1%)	0.404
Time from diagnosis to usage (months)		50.00 ± 40.52	64.45 ± 47.42	0.104
Time from usage to surgery (months)		22.03 ± 21.23		
Location at diagnosis				0.171
L1		4 (12.1%)	16 (9.8%)	
L2		6 (18.2%)	20 (12.3%)	
L3		13 (39.3%)	64 (39.3%)	
L4		2 (6.1%)	1 (0.6%)	
L3 + 4		0 (0%)	1 (0.6%)	
Unknown		8 (24.2%)	61 (37.4%)	
Behaviour at diagnosis				0.057
B1		14 (42.4%)	73 (44.8%)	
B2		4 (12.1%)	11 (6.7%)	
B3		6 (18.2%)	10 (6.1%)	
Unknown		9 (27.3%)	69 (42.3%)	

AZA/6-MP, azathioprine/6-mercaptopurine; L1, terminal ileum; L2, colon; L3, ileocolon; L4, upper GI; B1, non-structuring, nonpenetrating; B2, structuring; B3, penetrating.

**Table 3:** Multivariable analysis of the relationship between CD-related surgery and azathioprine/6-mercaptopurine treatment.

Dependent Variable	*n*		AZA/6-MP		*p* Value
	Surgery	No surgery	Odds ratio	95% CI	
Surgery (total)	462	699	0.094	0.070–0.127	<0.0001
Abdomen surgery	245	699	0.131	0.092–0.186	<0.0001
Perianal surgery	217	699	0.059	0.038–0.091	<0.0001

AZA/6-MP, azathioprine/6-mercaptopurine; CI, confidence interval.

**Table 4:** Multivariable analysis of the relationship between CD-related surgery and anti-TNF-α antagonists.

Dependent Variable	*n*		TNF-α Antagonist		*p* Value
	Surgery	No surgery	Odds ratio	95% CI	
Surgery (total)	462	699	0.225	0.151–0.335	<0.0001
Abdomen surgery	245	699	0.403	0.261–0.623	<0.0001
Perianal surgery	217	699	0.064	0.026–0.160	<0.0001

CI, confidence interval.
